# Understanding the effects of Cr doping in rutile TiO_2_ by DFT calculations and X-ray spectroscopy

**DOI:** 10.1038/s41598-018-26728-3

**Published:** 2018-06-07

**Authors:** G. Cristian Vásquez, David Maestre, Ana Cremades, Julio Ramírez-Castellanos, Elena Magnano, Silvia Nappini, Smagul Zh. Karazhanov

**Affiliations:** 1 Departamento de Física de Materiales, Facultad de CC. Físicas, Universidad Complutense, 28040 Madrid, Spain; 2 Department for Solar Energy, Institute for Energy Technology, 2007 Kjeller, Norway; 3 Departamento de Química Inorgánica I, Facultad de CC. Químicas, Universidad Complutense, 28040 Madrid, Spain; 4 IOM-CNR, Laboratorio TASC, S.S. 14-km 163.5, 34149 Basovizza, Trieste Italy

## Abstract

The effects of Cr on local environment and electronic structure of rutile TiO_2_ are studied combining theoretical and experimental approaches. Neutral and negatively charged substitutional Cr impurities Cr_Ti_^0^ and Cr_Ti_^1−^ as well as Cr-oxygen vacancy complex 2Cr_Ti_ + V_O_ are studied by the density functional theory (DFT) within the generalized gradient approximation (GGA) of Perdew-Burke-Ernzerhof (PBE) functional. Experimental results based on X-Ray absorption spectroscopy (XAS) and X-Ray photoelectron spectroscopy (XPS) performed on Cr doped TiO_2_ at the Synchrotron facility were compared to the theoretical results. It is shown that the electrons of the oxygen vacancy tend to be localized at the *t*_2*g*_ states of the Cr ions in order to reach the stable oxidation state of Cr^3+^. Effects of Cr on crystal field (CF) and structural distortions in the rutile TiO_2_ cell were analyzed by the DFT calculations and XAS spectra revealing that the CF and tetragonal distortions in TiO_2_ are very sensitive to the concentration of Cr.

## Introduction

Due to many technologically important optical, electronic and mechanical properties transition metal oxides have attracted increased research attention during the last years. Their optical, electronic and mechanical properties are appropriate for a wide range of applications. Titania (TiO_2_) is a semiconducting oxide that in both the anatase and rutile polymorphs have been applied in electronics, optoelectronics^1^, sensors^2^, photocatalysts^3,4^, energy production and storage^5^, etc. Study of cationic dopants in TiO_2_ is of great interest because it allows to modify and/or enhance physical and chemical properties of TiO_2_, thus advance the respective device performance and extend its application range^6–10^. Transition metal impurities such as Cr, V, Mn or Fe, can be easily incorporated into TiO_2_ lattice, which aroused interest since these dopants commonly improve physicochemical reactions at the surface that is important for degradation of pollutants in waste water treatment or in energy related applications where the oxidation state of the dopant plays a key role^6,8,11^. Nevertheless, achieving a controlled oxidation state of the transition metals in TiO_2_ is not a simple task, due to their characteristic multivalence. Many factors should be accounted for in the analysis of doping process, such as the temperature, atmosphere and precursors used during the synthesis, the dimensions and morphology of the material, and the final concentration of dopants, among others, which usually affect the position of the dopant in the lattice and its ground state nearby other intrinsic defects^11–13^. Among the other transition metals, Cr has frequently been the matter of study because of the controversy related to its influence on the physical and chemical properties of TiO_2_. As an example, Fan *et al*.^3^ observed that mesoporous Cr-doped TiO_2_ presents lower photocatalytic activity for acetaldehyde photodecomposition under the UV light radiation and its performance varies as a function of the Cr concentration and the irradiation wavelength, generally increasing below the critical Cr doping level. Similar observations were reported by Wei *et al*.^14^, Tian *et al*.^4^ On the contrary, Wilke *et al*.^6^ and recently Mittal *et al*.^15^ did not observe any photocatalytic improvement by Cr doping despite higher visible light absorption was reported. The magnetic properties of Cr doped TiO_2_ has been also investigated due to potential applications for spintronics and novel information processing^16^. In contrast to non-magnetic TiO_2_ observed by Matsumoto *et al*.^13^, ferromagnetic behavior at room temperature has been observed by other authors, however the variables involved in the magnetic properties are still unclear and remains under debate^9,16,17^. Therefore, a deeply understanding of electronic structure of doped systems like TiO_2_ is still needed, and the parameters such as the dopant oxidation state and the site where the dopant is located in the host lattice, as well as the influence of the dopant on the oxygen related defects should be further investigated in order to understand the dopant-induced physical and chemical effects^18^.

The present study focuses on modifications of electronic structure of rutile TiO_2_ induced by the incorporation of Cr, paying attention on the Cr oxidation state, location in the lattice, on formation of complex with oxygen vacancies, on its influence on lattice environment, and on its shallow ionization energy in the band gap. The aim of this work is combine theoretical and experimental studies of Cr-doped TiO_2_ samples, in order to shed light on the effects caused by doping in a wide range of concentrations that may affect their optoelectronic properties. Density functional theory (DFT) based calculations were carried out to investigate the effect of Cr on electronic density of states (DOS) of ideal and oxygen-deficient crystalline TiO_2_. The electronic structure has been experimentally studied by resonant and non-resonant X-ray Photoelectron Spectroscopy (XPS) and X-ray Absorption Spectroscopy (XAS) performed at a Synchrotron beamline that have been compared to the above theoretical results. The morphological and crystalline characteristics of the present microstructures, as well as the doping homogeneity achieved, make them an useful reference material for theoretical studies of Cr-doped TiO_2_. Moreover, this study could serve as a reference model for other metallic oxides that also crystallize in the rutile-like structure such as VO_2_, CrO_2_, MnO_2_, SnO_2_ or RuO_2_.

## Experimental Section

### Samples studied

The Cr-doped rutile TiO_2_ microtubes analyzed in this work have been synthesized by a vapor-solid method at temperatures of 1300 °C during 15 h, using 5 cat.% and 10 cat.% Cr-doped nanoparticles as precursor, as reported in a previous work^19^. These microtubes exhibit high crystallinity and chromium homogeneity, up to 3–4 cat.% Cr. A combined Raman spectroscopy in a confocal microscope and electron back scattered diffraction (EBSD) in a SEM^19^ confirm the growth direction and the lateral planes forming the tubes, which correspond to the [001] direction and the {110} family planes, respectively. These techniques also revealed the high crystalline quality of the microtubes, which lateral faces can be considered as single-crystals. In addition, undoped rutile TiO_2_ and Cr doped samples up to 5 cat.% Cr have been analyzed for comparison and commercial *α*-Cr_2_O_3_ polycrystalline powder (Sigma Aldrich, 99.9%), were employed as reference samples. Table 1 summarizes the samples used in this work and their Cr content.

**Table 1:** List of samples and corresponding Cr concentration in cationic fraction respect to Ti quantified by EDS.

Sample	Form	Contentration [cat.% Cr]
TiO_2_-np(R)	Nanoparticles	0.0
Cr02	Microtubes	1.8 ± 0.2
Cr03	Microtubes	2.8 ± 0.3
Cr04	Sintered	4.0 ± 0.3
Cr05	Nanoparticles	5.0 ± 0.4
Cr_2_O_3_ Ref.	Polycrystalline	—

### Characterization

XAS experiments in total electron yield (TEY) mode, as well as resonant and non-resonant XPS measurements were carried out at the BACH beamline at the Elettra Synchrotron light source facility (Trieste, Italy) using a monochromatic photon energy in the range from 400 to 600 eV, with energy resolution of 40–200 meV. The spectra were acquired in normal incidence geometry. C(1 s) peak at 284.6 eV from adventitious carbon^20^ has been employed for calibration of the XPS spectra. Chromium quantification was performed by energy dispersive spectroscopy (EDS) with a Bruker AXS 4010 detector mounted in a Leica 440 SEM.

### DFT Calculations

All the calculations were performed using the density functional theory (DFT) implemented in the Vienna *ab initio* simulation package (VASP)^21–23^ together with the potential projector augmented-wave (PAW) method^24–26^. The core and valence electronic states are expanded with a plane-wave basis set, with an optimal energy cutoff of 500 eV. Standard PAW-PBE^27^ pseudopotentials were employed to describe the Ti(3*s*3*p*3*d*4*s*), Cr(4*s*3*p*3*d*) and O(2*s*2*p*) valence states, allowing spin polarization during all calculations.

The defect calculations were performed using a 2 × 2 × 4 (96 atoms) supercell for the neutral and negative charged Cr defects (Cr_Ti_^0^, Cr_Ti_^1−^) and also for the combined neutral defect 2Cr_Ti_^0^ + V_O_^0^. A 4 × 4 × 6 mesh for the *k*-point sampling centered at the Gamma point was found optimal for the calculations. Additionally, calculations using hybrid functional with a 25% portion of Hartree-Fock exchange using the standard HSE06^28^ screening parameter of 0.2 Å^−1^ were performed to analyze the 2Cr_Ti_^0^ + V_O_^0^ case using a 2 × 2 × 2 supercell and a 2 × 2 × 2 mesh for the *k*-point sampling. The structural optimization for point defects in the supercell were performed starting from a pre-converged unit cell and allowing ionic relaxation with energy convergence of 10^−7^ eV per atom and minimizing the forces on all atoms less than 10^−2^ eV·Å^−1^. The calculation of the effective charges were performed using the Henkelman’s grid-based algorithm for the Bader electron decomposition method^29^. The effective charge is defined as *Q*_X_ = *Z*_X_-*q*_Bader,X_ (X = Cr, Ti, O) similar to that calculated in Vasquez *et al*.^30^, where *Z*_X_ is the number of valence electrons and *q*_Bader,X_ is the calculated Bader charge for the corresponding X atom. Graphical illustrations were drawn using the software VESTA^31^.

## Results and Discussion

### DFT study

Substitution of a single Ti atom by a Cr atom in the 2 × 2 × 4 rutile supercell corresponds to cationic concentration of ~3.1 cat.% Cr in good agreement with that measured by EDS in the studied Cr doped rutile microtubes^19^. The neutral Cr_Ti_ in ideal TiO_2_ lattice leads to Cr^4+^ ion, whereas the negatively charged Cr_Ti_^1−^ defect corresponds to Cr^3+^ ion. Both cases and the defect-impurity complex 2Cr_Ti_ + V_O_ are studied in this work and are compared to our experimental data obtained by XPS and XAS measurements.

The rutile structure is formed by an infinite chain of TiO_6_ octahedra with four basal (*d*_0_) and two apical (*D*_0_) Ti-O bond lengths. The basal bonds form an angle of 98.8° whereas the apical bonds form a right angle with respect to the basal plane^32^. Therefore, each octahedron is slightly tetragonal and trigonal distorted. In a previous work^30^ we have compared electronic properties of bulk rutile as calculated by PBE and hybrid functional (HSE06), as well as the effect of V_O_^2+^ on the TiO_2_ lattice and conduction band, so the complete list of rutile TiO_2_ parameters, as well as our experimental values and data from other authors, are summarized in the Table 2.

**Table 2:** List of lattice parameters, bond lengths, energy band gap and Bader effective charge calculated for the rutile phase.

	*a* [Å]	*c* [Å]	*d*_0_ [Å]	*D*_0_ [Å]	E_g_ [eV]	*Q*_Ti_ [|e|]	*Q*_O_ [|e|]
PBE^a^	4.65345	2.97300	1.96418	2.00662	1.8	+2.23	−1.12
HSE^a^	4.59042	2.95413	1.94500	1.98050	3.2	+2.43	−1.21
Experimental^b^	4.5937	2.9587	1.946	1.984	3.0–3.1		
PBE^c^	4.650	2.971		2.006	1.77	+2.22	−1.12
HSE^c^	4.590	2.947		1.980	3.05		
LDA^c^	4.557	2.929			1.79		

Comparison to experimental data and other theoretical studies are included.

^a^Previous work (ref.^30^); ^b^ref.^39,48–50^; ^c^ref.^33,44,51^.

Our results are in good agreement with those commonly reported by other authors. Small differences, below 1%, are found between the results obtained from the calculations obtained within PBE and HSE. Large differences are found in the calculated band gap (E_g_), which is underestimated by the PBE method. Janotti *et al*.^33^ reported that this effect in rutile TiO_2_ can be attributed to a reduced self-interaction for the O(2p)-derived states at the upper part of the valence band. Bader charge analysis calculated by HSE06 indicates a slightly higher ionic character in the Ti-O bond as compared with the PBE calculations^30^. Taking into account that, apart from E_g_ underestimation by the PAW-PBE funtional, comparable values are obtained by both computational methods, in this work the PBE method has been employed in most of the analyzed simulations, as it allows to spread the type of defects to be simulated while keeping calculation time lower than HSE06.

After the ionic relaxation we have observed that the symmetry of the CrO_6_ octahedron incorporated in TiO_2_ is different as compared to the TiO_6_ octahedron, which shows both tetragonal and trigonal distortions. In Table 3 the PBE calculated data for apical (*D*) and basal (*d*) Cr–O and Ti–O bond lengths in CrO_6_ and TiO_6_ octahedra are indicated for both Cr_Ti_^1−^ and Cr_Ti_^0^ defects in rutile TiO_2_.

**Table 3:** List of apical (*D*) and basal (*d*) bond length in CrO_6_ and TiO_6_ octahedra, |*D*−*d*|/*D* percentages, and Bader effective charge for Cr and Ti ions for Cr_Ti_^1−^ and Cr_Ti_^0^ defects in rutile.

	*D*_0_ [Å]	*d*_0_ [Å]	*|D−d|*/*D* [%]	*Q*_Cr,Ti_ [|e|]
**Cr**_**Ti**_^**1−**^				
CrO_6_	1.998	1.987	0.55	+1.75
TiO_6_	2.008	1.964	2.24	+2.23
**Cr**_**Ti**_^**0**^				
CrO_6_	1.939	1.943	0.21	+1.93
TiO_6_	2.009	1.965	2.24	+2.23

In addition, the corresponding (*D*-*d*)/*D* percentages and the calculated Bader effective charge values are also included in Table 3. In the case of the CrO_6_ octahedron, the tetragonal distortion is significantly reduced, where the relative difference between calculated apical (*D*) and basal (*d*) Cr–O bond length is less than 0.55% in comparison to the value obtained for Ti–O bonds of 2.4%. This observation indicates that the Cr ion symmetry in rutile TiO_2_ is nearly octahedral (*O*_*h*_) rather than tetragonal distorted octahedral symmetry as the TiO_6_ octahedron in perfect rutile.

**Figure 1:**
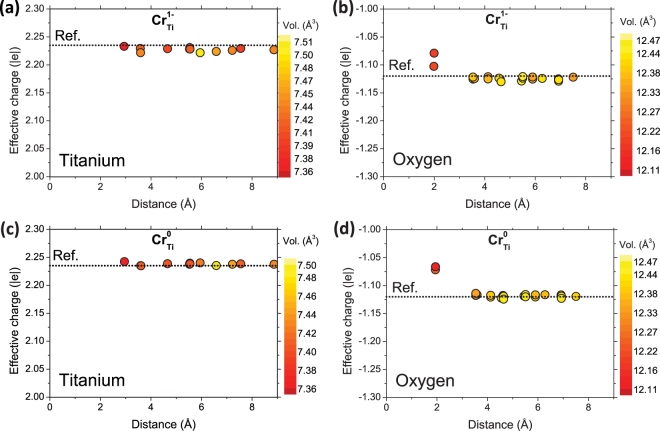
Bader effective charge as a function of the distance from the Cr defect for (**a**) Titanium atoms and (**b**) Oxygen atoms in Cr_Ti_^1−^ defect, and (**c**) Titanium atoms and (**d**) Oxygen atoms in Cr_Ti_^0^. Dotted line represents the Bader charge for bulk rutile.

Bader charge analysis as a function of the distance from the Cr_Ti_ defect, either for Cr_Ti_^1−^ or Cr_Ti_^0^, shows very small dispersion in effective charge values for O and Ti atoms as observed in the Fig. 1, which means that in both cases the electronic charge around the defect is very localized in the Cr atom. Only the nearest O atoms (ca. 2 Å), which are directly bonded to Cr, present small variations in the effective charge. Dots above the dotted line (reference from bulk TiO_2_) in Fig. 1(b,d) indicate that the O atoms coordinated with the corresponding Cr defect gain less charge than those O atoms only coordinated with Ti atoms in bulk rutile. Thus, the number of valence electrons in the different Cr defects (Cr_Ti_^1−^, Cr_Ti_^0^) influences the Cr-O bonding, as expected. The Cr *d*-orbital is occupied by two electrons in the case of a Cr^4+^ ion (Cr_Ti_^0^ defect), and three in the case of a Cr^3+^ ion (Cr_Ti_^1−^). This implies that the additional electron in the Cr_Ti_^1−^ defect belongs to the Cr valence electrons, expanding slightly the octahedron volume by increasing the Cr-O bond length (see Table 3).

**Figure 2:**
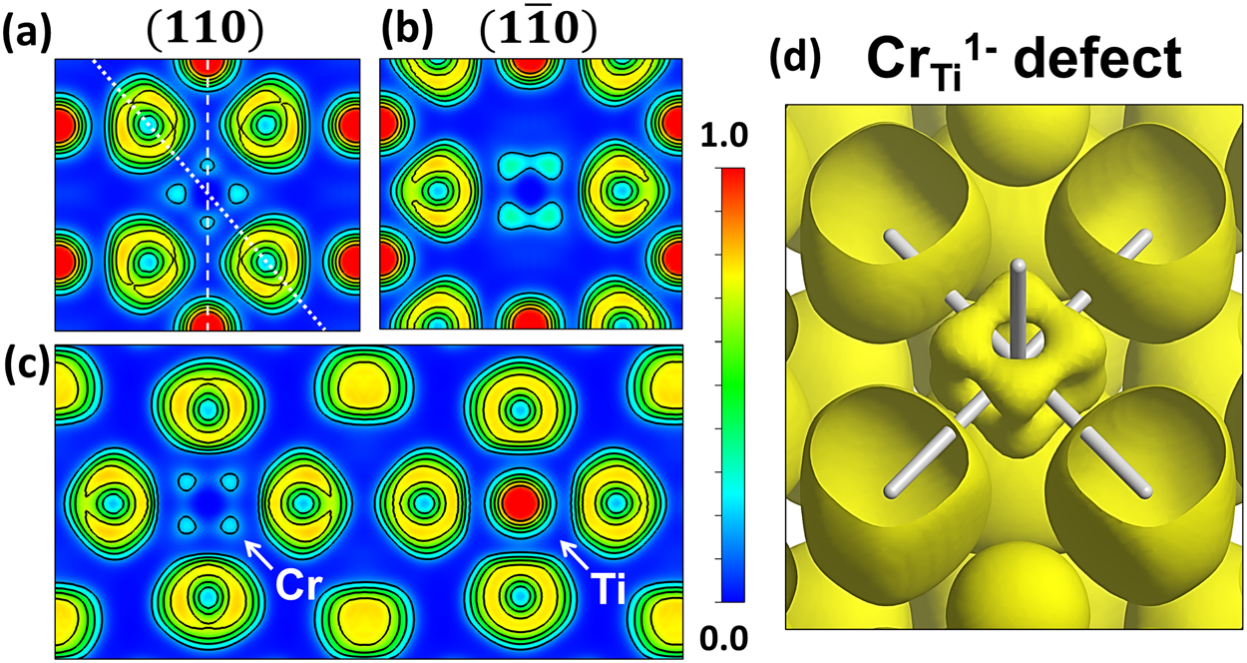
ELF cross section along the (**a**) (110) plane, (**b**) ($1\bar{1}0$) plane and (**c**) the apical plane perpendicular to (110) for the Cr_Ti_^1−^ defect. Dotted line in (a) represents the intersection with ($1\bar{1}0$) plane and dashed line represents the intersection with the apical plane. (**d**) Isosurface for an ELF = 0.15 for the Cr_Ti_^1−^ defect. Cr–O bonds are represented as white sticks.

**Figure 3:**
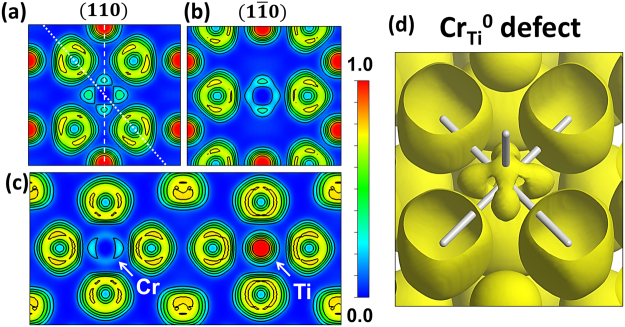
ELF cross section along the (**a**) (110) plane, (**b**) ($1\bar{1}0$) plane and (**c**) the apical plane perpendicular to (110) for the Cr_Ti_^0^ defect. Dotted line in (**a**) represents the intersection with ($1\bar{1}0$) plane and dashed line represents the intersection with the apical plane. (**d**) Isosurface for an ELF = 0.15 for the Cr_Ti_^0^ defect. Cr–O bonds are represented as white sticks.

More differences can be appreciated by the observation of the electronic distribution by means of the electron localization function (ELF), corresponding to both types of Cr defects. In Fig. 2(a,b) the ELF basal cross-section planes for the rutile (110) and ($1\bar{1}0$) planes, respectively, are represented for the case of Cr_Ti_^1−^. The ($1\bar{1}0$) plane is perpendicular to (110) plane crossing along the dashed line marked in Fig. 2(a), where the Cr_Ti_^1−^ defect is located at the center. Figure 2(c) corresponds to the apical plane crossing along the dotted line of the Fig. 2(a). The equivalent cross-section planes corresponding to the Cr_Ti_^0^ defect in rutile TiO_2_ are represented in Fig. 3.

In both cases the electron density around the basal Cr-O bonds [Figs 2(a) and 3(a)] is practically negligible in the (110) planes, and in a greater extent for the Cr_Ti_^1-^ defect. However, the apical bonds are different depending on the Cr oxidation state. For the neutral Cr_Ti_^0^ defect (Cr^4+^) the ELF value is higher in the apical Cr–O bonds, contrary to Cr_Ti_^1−^ (Cr^3+^) where the electron density is still minimum as can be observed in Figs 2(b) and 3(b), corresponding to the ($1\bar{1}0$) planes. The trigonal distortion in the (110) plane modifies the electron density, thus increasing the ELF value in the regions where O–Ti–O angle is >90°, unlike the apical plane represented in the Figs 2(c) and 3(c) where all the Cr–O bonds are nearly equivalent and the plane is unaffected by octahedral distortions. The isosurfaces for an ELF value of 0.15 corresponding to the Cr_Ti_^1−^ and Cr_Ti_^0^ defects are shown in the Figs 2(d) and 3(d), respectively. The Cr–O bonds are represented as white sticks. All the features described for the ELF cross-sectional views can be appreciated in the volumetric space in Figs 2(d) and 3(d). In this way, the electron density around Cr_Ti_^1−^ defect is distributed forming a cube-like volume, whereas the electron localization around the Cr_Ti_^0^ defect shows a more complex distribution with localized electrons in the regions around the apical bonds. The relatively small difference in the effective charges between the Cr_Ti_^1−^ and Cr_Ti_^0^ defects (Table 3) combined with the ELF results suggest that the Cr–O bonding character is mostly ionic in the case of a Cr_Ti_^1−^ defect, whereas the apical Cr–O bonds in the Cr_Ti_^0^ defect present a covalent character slightly higher than Ti^4+^ in TiO_2_. Thereby, the different number of valence electrons affects differently to the electron distribution in each Cr defect. The characteristic ELF of the Cr_Ti_^1−^ defect could be explained as valence electrons that tend to occupy the ${d}_{{xy}}$, ${d}_{{yz}}$ and ${d}_{{zx}}$ orbitals, pointing to directions between Cr–O bonds, instead of the ${d}_{{z}^{2}}$ and ${d}_{{x}^{2}-{y}^{2}}$ orbitals which are pointing towards the coordinated O atoms, This suggests that the electron configuration in the ground state of Cr_Ti_^1−^ defect is 3*t*_2*g*_^3^*e*_*g*_^0^ characteristic of an Cr^3+^ ion under octahedral (*O*_*h*_) crystal field. In the case of the Cr_Ti_^0^, the higher electron localization along the apical bonds indicates that the electrons can partially occupy the ${d}_{{z}^{2}}$ orbitals.

**Figure 4:**
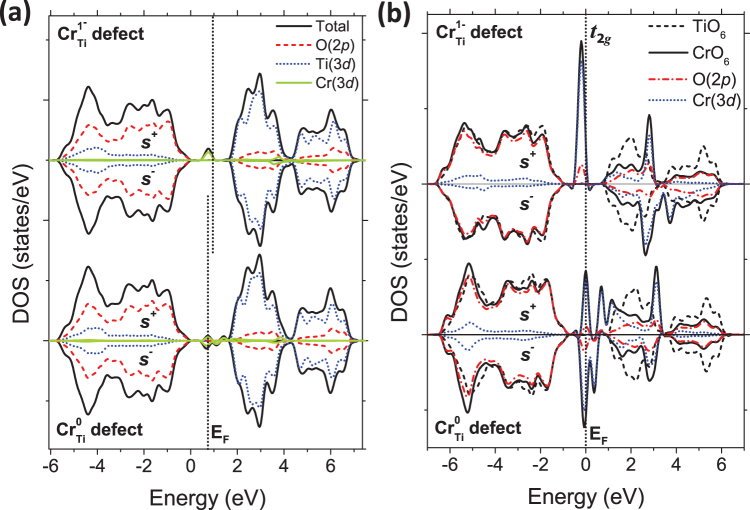
(**a**) Spin polarized (*s*^*+*^, *s*^*−*^) total DOS and pDOS corresponding to O(2*p*), Ti(3*d*) and Cr(3*d*) states for Cr_Ti_^1−^ and Cr_Ti_^0^ defects, and (**b**) DOS contributions from CrO_6_ and TiO_6_ (dashed) octahedra.

In order to complete the DFT study, the density of electronic states (DOS) has also been analyzed. The total DOS and partial DOS (pDOS), including O(2*p*), Ti(3*d*) and Cr(3*d*), for the Cr_Ti_^1−^ and Cr_Ti_^0^ defects in rutile TiO_2_ are shown in Fig. 4(a). For clarity, the VB maximum (VBM) is set to 0 eV, and *s*^+^ and *s*^-^ indicate the spin-up and spin-down components of the DOS, respectively.

The pDOS reveals that the Cr defect states within the TiO_2_ band gap are originated by orbitals of Cr 3*d* nature. With regard to the Cr_Ti_^1−^ defect [top of the Fig. 4(a)], the occupied electronic states are localized as a spin-up level 0.2 eV below the E_F_ and about 0.75 eV above the VBM. The neutral Cr_Ti_^0^ defect [down in the Fig. 4(a)] shows electronic states partially occupied within the E_g_ with a symmetric state below the E_F_ and a more complex defect structure with spin-up and spin-down polarized states at the CB region. Figure 4(b) shows the partial contribution of O(2*p*) and Cr(3*d*) orbitals for CrO_6_ octahedron compared with a single TiO_6_ octahedron (dashed line) for both Cr_Ti_^1−^ and Cr_Ti_^0^. In the case of Cr_Ti_^1−^ [top of the Fig. 4(b)] the calculated VB contributions of the CrO_6_ octahedron are similar to the calculated contributions for the TiO_6_ octahedron. Within the band gap region, the O(2*p*) orbitals are slightly hybridized with Cr(3d) orbitals (*t*_2*g*_ related states) in the Cr_Ti_^1−^ defect. On the contrary, for the Cr_Ti_^0^ defect [down in Fig. 4(b)], the O(2*p*) and Cr(3*d*) orbitals show stronger hybridization states at about 5.2 eV below the E_F_ partially overlapped with the O(2*p*)-*σ* bonds of TiO_2_^34^. In the conduction band (CB) the Cr contributions are partially mixed with the empty *t*_2*g*_ states of the TiO_2_ CB^33^, showing localized states at about 3 eV above the E_F_. Hereinafter our analysis will be focused on the VB region, thus results comparing the PAW-PBE and HSE06 functional are shown in Supplementary Fig. S1 confirming that the VB region are qualitatively similar using both functionals.

**Figure 5:**
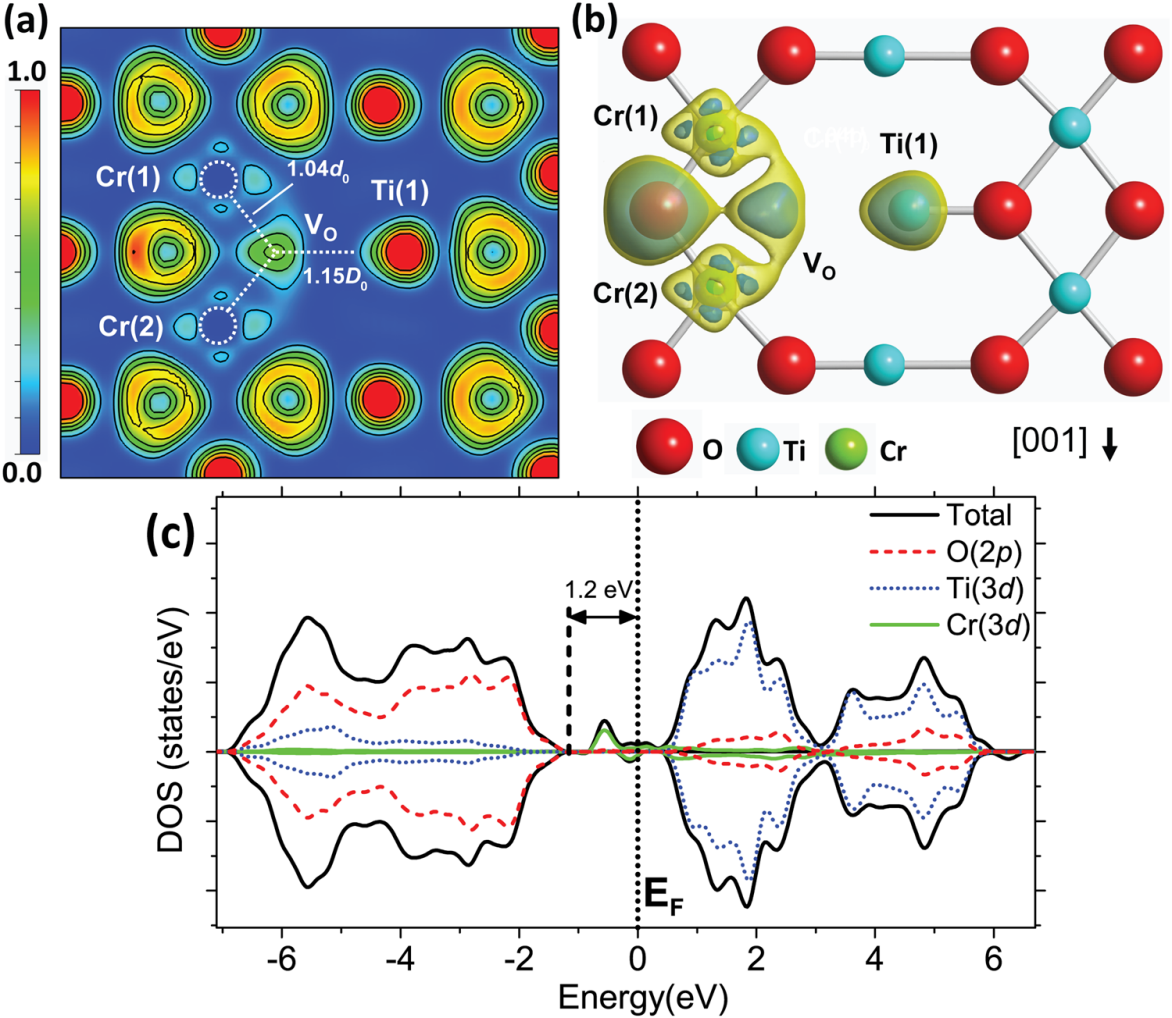
(**a**) ELF cross section along the (110) plane with a combined 2Cr_Ti_^0^ + V_O_^0^ defect. Dotted circles and lines represent the Cr atoms and dangling bonds, respectively. (**b**) ELF isosurfaces for an ELF value of 0.11 (yellow/light) and 0.21 (blue/dark) around the nearest Cr, Ti and O atoms from the V_O_ defect. (**c**) Spin polarized (*s*^*+*^, *s*^*−*^) total DOS and pDOS corresponding to O(2*p*), Ti(3*d*) and Cr(3*d*) states for the combined 2Cr_Ti_^0^ + V_O_^0^ defect.

Other way to obtain Cr^3+^ defects in a neutral rutile lattice could be achieved by the electron transfer from neutral oxygen vacancies, V_O_^0^, or ionized V_O_^*n*+^ defects (*n* = 1, 2), to nearby Cr^4+^ defects. To perform and simulate this possibility, a single V_O_^0^ is created in the proximity of two Cr_Ti_^0^ defects resulting in the combined defect 2Cr_Ti_ + V_O_ studied in this work. Figure 5(a) shows the ELF cross section for the (110) plane calculated by PBE for the combined defect 2Cr_Ti_ + V_O_. The dotted lines indicate broken Cr–O and Ti–O bonds due to the removed oxygen atom (V_O_).

After ionic relaxation, the trapped electrons from the removed O atom are localized in the region between the Cr and Ti dangling *d* orbitals. The presence of the V_O_ defect generally results in displacements of the Ti and O atoms outward and inward, respectively, from the V_O_ site^30,33,35^ In the combined defect, the Cr atoms [Cr(1) and Cr(2) in Fig. 5(a)] are displaced outward from the V_O_^0^, but only a small amount (1.04*d*_0_) as compared to the nearest Ti atoms from the V_O_^2+^ defect (1.16*d*_0_ from Janotti *et al*.^33^). However, the next nearest Ti atom [Ti(1) in Fig. 5(a)] is displaced an amount of about 1.15*D*_0_, equivalent to that calculated for V_O_^2+^ (1.15*D*_0_ from Janotti *et al*.^33^). Despite the fact that charge density in the bonded O atom [between Cr(1) and Cr(2) in the Fig. 5(a)] is repelled outward from the vacancy site, observed as a high electron localization in the opposite side from the V_O_, only small distortions can be observed around Cr atoms because of the ionic relaxation as compared to single V_O_^2+^. The isosurfaces represented in the Fig. 5(b) for ELF values of 0.11 (in yellow/light) and 0.21 (in blue/dark) show that electron localization geometry around Cr atoms is similar to that observed for the Cr_Ti_^1−^ defect [Fig. 2(d)]. Therefore, the electron localization is also characteristic for Cr^3+^ ions. However, in this case the electrons from the V_O_ state are bounded with Cr atoms indicating the presence of a mixed Cr(3*d*)–V_O_ electronic state. The Total DOS and pDOS corresponding to O(2*p*), Ti(3*d*) and Cr(3*d*) states for the combined defect 2Cr_Ti_ + V_O_ are represented in Fig. 5(c). It can be noticed that DOS shape is similar to that calculated for the Cr_Ti_^1−^ defect [top in Fig. 4(b)] characterized by spin-up polarized defect states of Cr(3*d*) nature at about 0.75 eV above the VBM. For the combined defect there are also partially occupied states close to the E_F_ which increase the energy splitting between the VBM and the E_F_ from 0.95 eV, for Cr_Ti_^1−^, to 1.2 eV, for the combined 2Cr_Ti_ + V_O_. In this case the electronic states of the V_O_^0^ are mixed with the nearest Cr *t*_2*g*_ orbitals resulting in a high spin polarized state, similar to that observed for the Cr_Ti_^1−^, which means that electrons from V_O_ contribute to the formation of a pair of Cr^3+^ defects.

### XPS and XAS study

**Figure 6:**
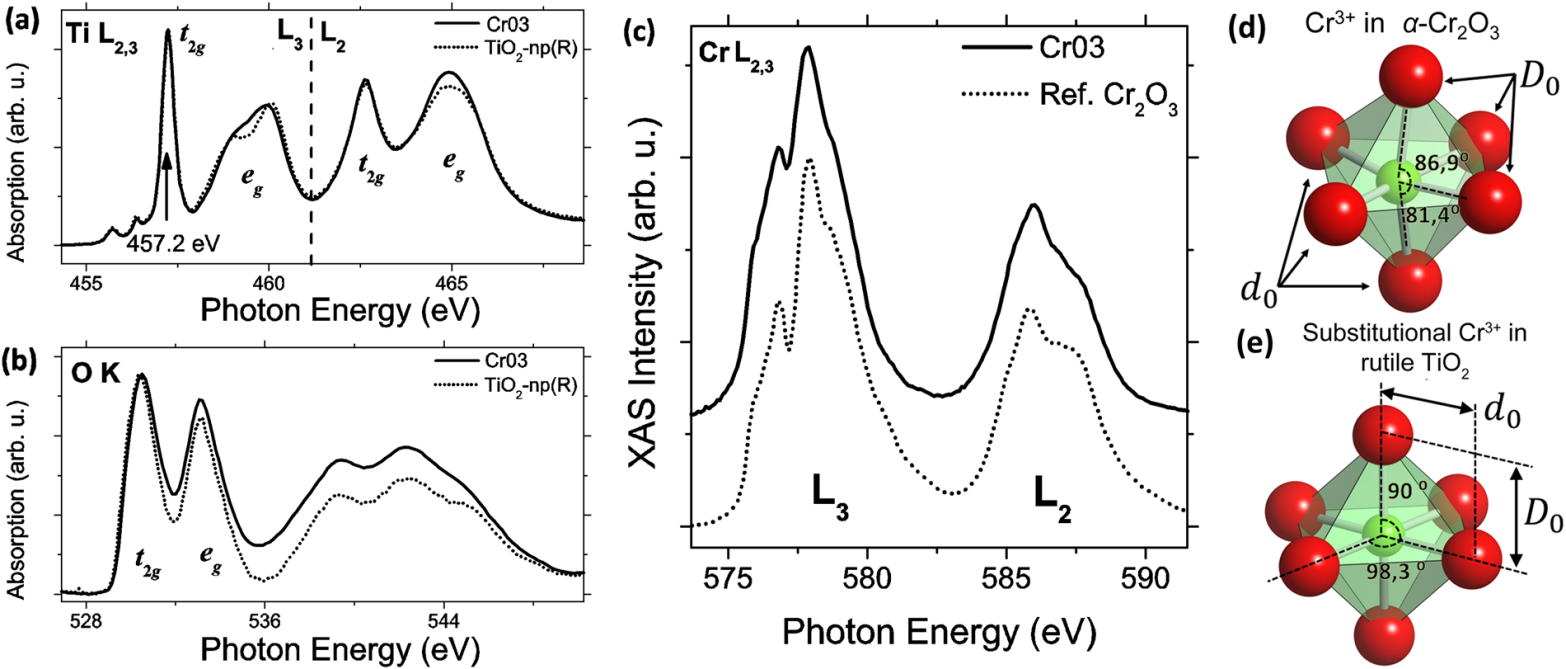
(**a**) XAS spectra from Ti-L_2,3_ edge, (**b**) O-K edge and (**c**) Cr-L_2,3_ edge. (**d**) CrO_6_ octahedron from *α*-Cr_2_O_3_ and (**e**) Cr_Ti_^1−^ defect in rutile TiO_2_.

XAS and XPS measurements have been performed at BACH beamline at the Elettra synchrotron facility in order to investigate the electronic structure and the effect of Cr in rutile TiO_2_. The combination of these experimental results with the theoretical study will extend the understanding of the Cr doping process in rutile TiO_2_. In this way, XAS spectra have been acquired on the Cr doped microtubes, as well as on undoped TiO_2_ and Cr_2_O_3_ reference samples. The Fig. 6(a–c) show the XAS spectra from Ti-L_2,3_, O-K and Cr-L_2,3_ absorption edges, respectively, from the doped Cr03 microtubes. Spectra from undoped TiO_2_ and Cr_2_O_3_ are also included for comparison. The Ti-L_2,3_ edge is splitted in two regions due to the spin-orbit coupling forming the L_3_ and L_2_ edges, as marked in Fig. 6(a).

Both L_3_ and L_2_ bands are subdivided in two contributions as a result of the crystal field (CF), that originates the fivefold *d*-orbital splitting into two degenerated levels of *t*_2*g*_ (three-fold degenerate states) and *e*_*g*_ (two-fold degenerate states) symmetry by an energy amount of 10 *Dq* eV, also known as CF splitting^36^. According to the atomic coordinates calculated by DFT, TiO_6_ octahedron in the rutile phase presents both trigonal and tetragonal distortions. TiO_2_ tetragonal distortions, affecting directly to apical Ti–O bonds, are more sensitive in the L_2,3_-edge. Under tetragonal distortion the *e*_*g*_ states are splitted in two states of *b*_1*g*_ and *a*_1*g*_ symmetry, whereas *t*_2*g*_ states are splitted in three states of *b*_2*g*_ and *e*_*g*_ (two-fold degenerated) symmetry^37^. The high energy resolution achieved in the acquisition of the XAS spectra (<0.1 eV) enables the observation of the *e*_*g*_ splitting at the L_3_ edge, resulting in the characteristic rutile TiO_2_ fingerprint with a maximum at 460 eV and a shoulder around 459 eV^32,37^, as observed in Fig. 6(a). Analogously, this association can be extended to the L_2_ edge, although the absorption bands in the latter region are broader because of Auger decay effects and vibrational dispersions^38^. It can be noticed that in Fig. 6(a) the *e*_*g*_ band presents slight, but not negligible, modifications in Cr doped samples such as a less defined *e*_*g*_ splitting at the L_3_ edge or variations in the *e*_*g*_ relative intensity at the L_2_ edge.

On the other hand, the O-K edge [Fig. 6(b)], corresponding to electronic transition 1*s* → 2*p*, also shows features that are sensitive to the crystal environment. The hybridization of the Ti(3*d*)–O(2*p*) orbitals in TiO_2_ makes able the observation of the *t*_2*g*_ and *e*_*g*_ bands in the O-K edge^32^, observed at 530 and 533 eV, respectively. Complex contributions at higher energies with two maxima at 539 and 542 eV and a shoulder at around 545 eV, are also characteristic of the rutile phase of TiO_2_^39^. In this case variations related to the *e*_*g*_ relative intensity can be observed in Fig. 6(b), similar to that observed at the L_2_ edge in Fig. 6(a).

The Cr L_2,3_ edge [Fig. 6(c)] shows a more complex structure. The number of allowed transitions is significantly greater for the Cr atom due to the partially filled d orbitals in the case of Cr^3+^ or Cr^4+^ oxidation states, making difficult the analysis of the Cr-L edge. Thus, the Cr-L_2,3_ edge from an *α*–Cr_2_O_3_ sample has been used as a reference and compared to the Cr-L_2,3_ from Cr03 microtubes. The main features in the corresponding XAS spectra in Fig. 6(c), as the maxima at 576.6 and 577.5 eV, or the spin-orbit energy splitting (L_3_–L_2_ separation), indicate that the most probable oxidation state in the Cr doped TiO_2_ microtubes is Cr^3+^ under octahedral coordination, in agreement with previous CL results^11,19^. However, the characteristic spectral features could be associated with the different octahedral distortions or local environment, as can be observed from the ball and stick diagrams for a CrO_6_ unit from *α*–Cr_2_O_3_ [Fig. 6(d)] and the calculated CrO_6_ octahedron for a Cr^3+^ defect in rutile TiO_2_ [Fig. 6(e)].

**Figure 7:**
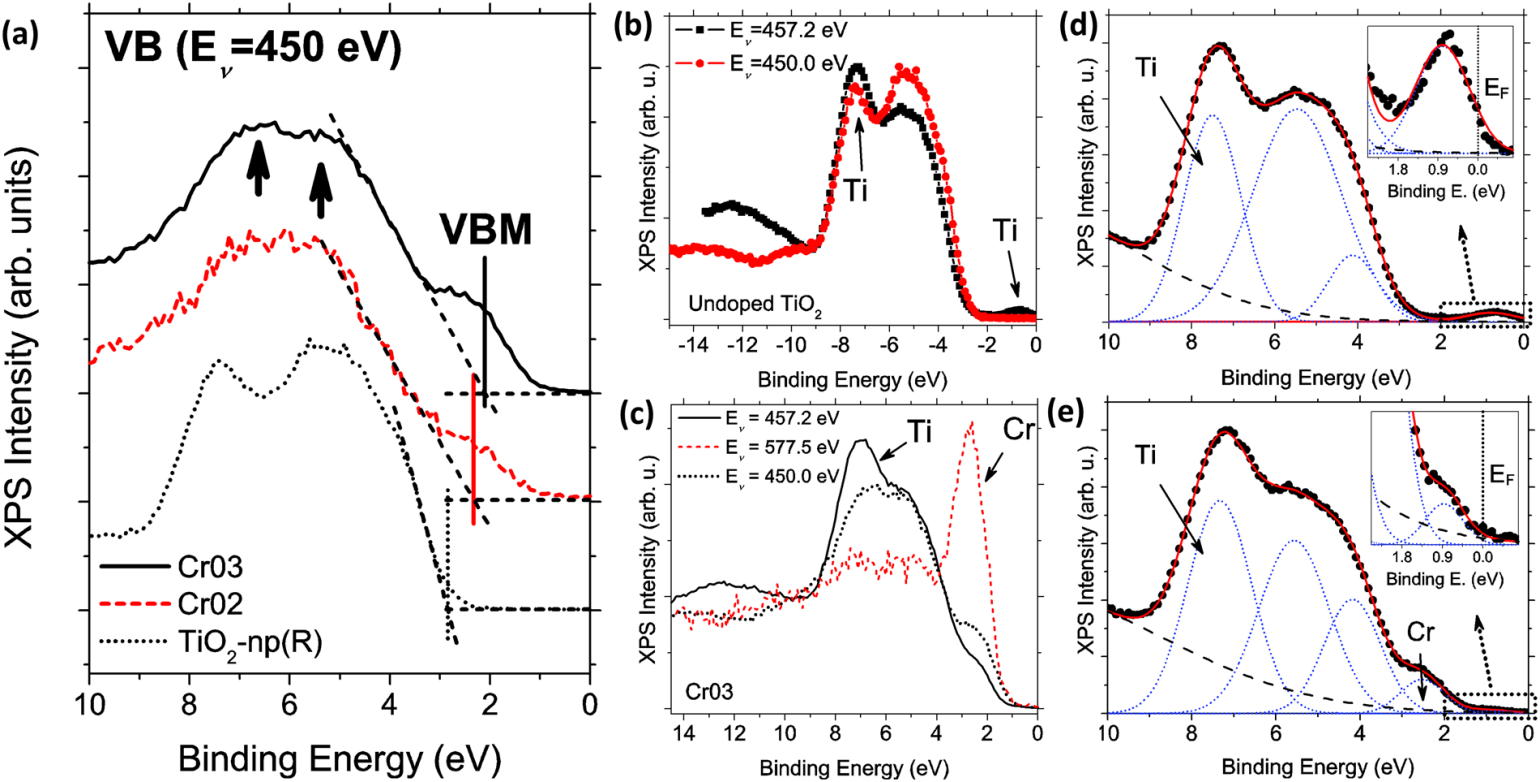
(**a**) VB-XPS spectra from undoped (TiO_2_-np(R)) and Cr doped TiO_2_ with different Cr concentration (Cr02, Cr03) using a photon energy E_*ν*_ = 450 eV. The dashed lines are indicative for the estimation of the VBM. (**b**) On and off-resonance BV spectra from undoped TiO_2_ and (**c**) Cr-doped TiO_2_ microtubes (Cr03). Arrows in (**b**) and (**c**) mark the resonant peaks according to the absorption maximum selected. (**d**) Gaussian deconvolution (straight line) of Ti resonant XPS spectra (dots) measured with a photon energy of 457.2 eV for TiO_2_-np sample, and (**e**) Cr doped TiO_2_ (Cr03). The insets in (**d**) and (**e**) correspond to enlargement of the E_F_ region.

XPS spectra at the VB region (VB-XPS) from microtubes with different amount of Cr (see Table 1 in Experimental Section) have been analyzed, in addition to an undoped TiO_2_ sample used as a reference. VB-XPS spectra acquired with energy of 450 eV are shown in Fig. 7(a).

Changes in the VB region can be observed as a function of the Cr doping. The undoped sample used as reference [dotted line in Fig. 7(a)] shows a VB-XPS spectrum with a broad band centered at 6 eV composed by two contribution with maxima located at 7.4 and 5.3 eV.

These contributions, attributed to O(2*p*)–*σ* and O(2*p*)–*π* states that are partially hybridized with Ti(3*d*)^34,40^, can be also observed, although less defined, in the Cr doped samples Cr02 and Cr03 [marked with arrows in Fig. 7(a)]. In Cr doped TiO_2_, the VB is composed by a broad band, wider than that from undoped TiO_2_, centered at about 6 eV that extends approximately from 9 to 1 eV. Moreover, a shoulder close to the VBM, around 2.5–2.6 eV, is observed differently from the undoped TiO_2_. This band can be attributed to Cr(3*d*) states^10^, as it is higher as the amount of Cr increases. At the same time, the VBM shifts towards the E_F_ as the amount of Cr increases in the microtubes, which should involve a less *n*-type behavior because of Cr doping at the surface of the probed microtubes.

Resonant XPS helps for the identification of valence states from Ti and Cr species. Figure 7(b) and (c) show the VB-XPS spectra acquired using photon energies corresponding to absorption maxima measured for Ti and Cr, according to their L_2,3_ absorption edge spectra [Fig. 6(a,c)]. Figure 7(b) shows the VB spectra for undoped TiO_2_ (TiO_2_-np) using a photon energy off-resonance (E_*ν*_ = 450 eV) and on-resonance (E_*ν*_ = 457.2 eV). The XPS signal from Ti states in the VB increases under on-resonance conditions, showing peaks at 7.3 eV and 0.7 eV below E_F_, marked with arrows in Fig. 7(b). The photoemission band at 7.3 eV is associated to hybridized O(2*p*)–Ti(3*d*) *σ*-bonding states^34,40^, whereas the band at 0.7 eV is associated to Ti(3*d*) states as the result of reduced Ti^3+^ ions due to presence of oxygen vacancies and structural defects^33,35^. The same experiment was carried out for Cr doped microtubes (Cr03), as shown in Fig. 7(c). In this case, a peak with a maximum at 7.0 eV is observed by using a photon energy on-resonance for Ti (E_*ν*_ = 457.2 eV) which can be also related to O(2*p*)–Ti(3*d*) hybridized states. However, selecting a photon energy on-resonance for Cr (E_*ν*_ = 577.5 eV) a peak at 2.7 eV clearly dominates the VB spectrum. This band at 2.7 eV was previously observed as a shoulder near the VBM in all the VB-XPS spectra measured for Cr doped samples [Fig. 7(a)], confirming that this contribution is related to Cr(3*d*) states^10^.

Figures 7(d,e) show the Gaussian deconvolution of the VB spectra in Fig. 7(b,c) acquired on-resonance for Ti atoms for undoped TiO_2_-np and Cr03 samples, respectively. The insets of the Fig. 7(d,e) show enlargement of the E_F_ region. In the undoped sample the signal associated with Ti^3+^ defects at 0.7 eV below E_F_ is clearly observed, indicating higher concentration of these defects in comparison to Cr doped TiO_2_, in which the signal is practically negligible as shown in the Fig. 7(e). However, analyzing in detail the VB spectra of Cr doped samples [inset if Fig. 7(e)], the presence of Ti^3+^ defects can be observed on-resonant conditions as a very weak band. This can explain the observed E_F_ shift towards the VBM [Fig. 7(a)] as a result of Cr doping, which indicates that a less n-type character can be induced by controlling the Cr concentration^41^.

### Analysis and comparison to DFT calculations

**Figure 8:**
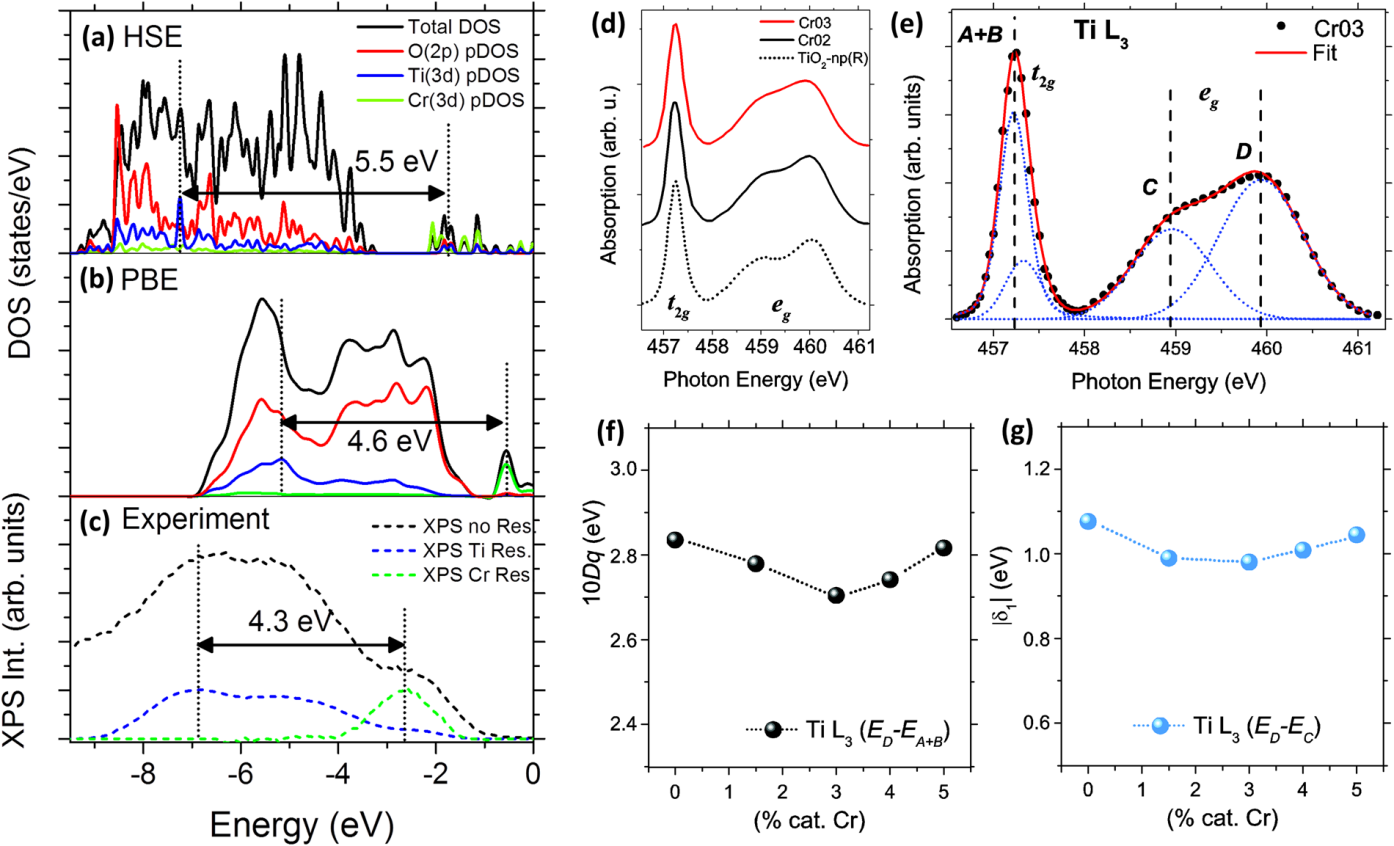
Comparison of the calculated DOS for 2Cr + V_O_ defect calculated by (**a**) HSE functional, (**b**) PBE and (**c**) the experimental VB spectrum (Cr03 sample). The intensity of the on-resonance spectra in (**c**) are reduced to obtain similar features. (**d**) Ti-L_3_ spectra from undoped and Cr doped TiO_2_ samples (Cr02 and Cr03). (**e**) Example of a Gauss-Lorentz (G-L) deconvolution of the Ti-L_3_ edge. (**f**) Estimation of 10*Dq* parameter as a function of the Cr concentration from band deconvolution of Ti-L_3_ edge. (**g**) Tetragonal distortion parameter |*δ*| as a function of the Cr concentration.

Theoretical DFT calculations and experimental VB-XPS results acquired on Cr doped TiO_2_ have been compared, as shown in Fig. 8(a–c), in which the calculated DOS have been compared to the experimental results from Cr03 sample, and the intensity of the resonant Ti and Cr contributions have been adjusted to fit with the off-resonant VB spectrum.

Among the considered defects, the best fit between experimental VB and calculated data is achieved for the 2Cr + V_O_ defect using the PBE functional. In the calculated combined defect the difference between the pDOS maxima related to Ti(3*d*) and Cr(3*d*) states is about 5.5 eV for HSE [Fig. 8(a)], 4.6 eV for PBE [Fig. 8(b)], whereas the experimental difference between the Ti and Cr resonant peaks is about 4.3 eV [Fig. 8(c)]. Thus, the Cr defect states of 3*d* nature are located within the E_g_ of TiO_2_. The deviation between the Ti(3*d*)-Cr(3*d*) states separation calculated by DOS and experimental VB may be originated by the resultant structural relaxation of the Cr defects^42^, as well as the differences between the DFT model and the measurement conditions of the actual system.

Oxygen defects and other structural defects, such as Ti interstitial, can be found naturally in a real TiO_2_ system, and more frequently at the surface^33,35,43^. According to our DFT results, in order to achieve charge neutrality, Cr atoms could trap electrons from V_O_^0^ defects and hence be reduced to the most stable oxidation state (Cr^3+^). Therefore, two Cr defects could be compensated by the creation of a single V_O_^0^. The presence of Ti^3+^ defects are related to the presence of V_O_^0^, in which two electrons can occupy Ti(3*d*) states reducing two nearby Ti^4+^ ions^33,44^. Kim *et al*.^45^ stated that Cr^3+^ defects in rutile TiO_2_ are promoted when the samples are grown under oxygen poor conditions and determined that the formation energy of V_O_^2+^ and Cr^3+^ is smaller than the formation energy of Cr^4+^. Taking into account that the formation energy of V_O_^2+^ is also smaller than V_O_^1+^ and V_O_^0 ^^33,35^, it is expected that the preferential oxidation state of Cr in TiO_2_ should be Cr^3+^. Thus, a single V_O_^0^ could be changed into V_O_^2+^ by the reduction of Cr^4+^ ions into Cr^3+^, leading to higher concentration of coordinated Cr^3+^–Vo^2+^–Cr^3+^ defects and hence reducing the presence of Vo^0^. This is consistent with the observed quenching of the Ti^3+^ related emissions in the luminescence spectra of Cr doped TiO_2_ favoring the Cr^3+^ luminescent emissions, reported in previous works^11,19^. However, experimental VB spectrum shows that the E_F_ location is about 2.6 eV above the Cr resonant peak, which means that, considering that the E_g_ of crystalline rutile is about 3.1 eV, the E_F_ localization is still close to the CB of rutile TiO_2_.

The effect of the crystal field (CF) is directly related to the coordination symmetry of Ti atoms, which implies that the hybridized metal-ligand bonds (O(2*p*) orbitals in TiO_2_) are very sensitive to local distortions in the rutile lattice^32^. A complementary study on the CF splitting and tetragonal distortions associated with the presence of Cr in rutile TiO_2_ has been also performed by a deeper analysis of the Ti-L_3_ absorption edge. The tetragonal distortion, which is due to the elongation of the apical Ti–O bonds, has a strong influence on the Ti-L_2,3_ edge features rather than trigonal distortions, which can be neglected in TiO_2_^36,38^. This implies that a simplified *D*_4*h*_ symmetry is enough to describe the Ti^4+^ ion in the rutile cell^38^. Figure 8(d) shows the Ti-L_3_ edge for undoped TiO_2_ and Cr-doped TiO_2_ microtubes (Cr02 and Cr03), where slight variations can be appreciated as the concentration of Cr increases. As aforementioned, Cr incorporation modifies slightly the L_3_ edge, and in a greater extent the *e*_*g*_ related band. In this case, by Gauss-Lorentz (G-L) band deconvolution, the L_3_ edge can be decomposed into four bands labeled as *A*, *B*, *C* and *D*. It can be observed in Fig. 8(e) that *A* and *B* bands are nearly overlapped for *t*_2*g*_ states. Thereby, *t*_2*g*_ states are identified by the *A *+* B* maximum. *C* and *D* bands, located at higher energies and related to the *e*_*g*_ states, show an absolute energy difference |*E*_*C*_ − *E*_*D*_| of about 1 eV. Information related to the crystal environment of Ti ions can be extracted from the G-L deconvolution of experimental Ti L_3_ edge, in which *E*_*D*_ − *E*_*A*+*B*_ could be related to the variations of the CF splitting (10*Dq*) and the difference |*E*_*C*_ *–* *E*_*D*_| could be associated with variations of the tetragonal distortion parameter defined in absolute value as |*E*(*b*_1*g*_) − *E*(*a*_1*g*_)| = |*δ*|^38^.

Figures 8(f,g) show an estimation of the CF splitting (10*Dq*) and the |δ| parameter as a function of the amount of Cr, in cationic fraction, calculated from the Ti-L_3_ absorption edge corresponding to samples Cr02, Cr03 and samples with variable content of around 4 cat.% and 5 cat.% Cr, composed by sintered grains and precursor powder respectively. In both cases, the CF splitting decreases when Cr dopants are incorporated up to 3 cat.% Cr, and tends to increase when the amount of Cr is above this value. According to our previous results, the Cr solubility limit observed for rutile TiO_2_ microtubes grown by vapor-solid method is in the order of 3 cat.% Cr, which could indicate that the Cr defect site in the rutile lattice varies for concentrations either below or above this value. The |*δ*| parameter, associated with tetragonal distortion, also presents a slight reduction up to 3 cat.% Cr. Analyzing the calculated atomic coordinates of the Ti and O atoms surrounding the Cr_Ti_^1−^ defect, a tetragonal distortion reduction has been observed in some of the TiO_6_ octahedra. The apical (*D*) and basal (*d*) Ti–O bonds of the in-plane TiO_6_ octahedra which are sharing corners with the CrO_6_ octahedron are D = 2.000 Å and d = 1.967 Å respectively, so the relative difference |*D* − *d*|/*D* is reduced from 2.4% (bulk rutile TiO_2_) to 1.6% using the calculated *D* and *d*, indicating a reduction of the tetragonal distortions. This is in agreement with the reduction of the |*δ*| parameter calculated by the G-L deconvolution of experimental Ti-L_3_ spectra.

As the local distortions induced by Cr affects directly to the crystal environment of TiO_2_, the physical behavior of the Cr defects should be also dependent on their local environment. The relatively small tetragonal distortion calculated for both Cr_Ti_^0^ and Cr_Ti_^1−^ defects indicates that the CrO_6_ octahedra in TiO_2_ present *O*_*h*_ symmetry independently of the Cr oxidation state. XAS, XPS and DFT results indicate a predominant Cr^3+^ ion in our system. Previous work^19^ reported a characteristic emission related to Cr^3+^, also called *R*-lines, using CL spectroscopy as an evidence of the presence of octahedral coordinated Cr^3+^ ions in our Cr-doped TiO_2_ microtubes. However, that emission was observed at low temperature (T = 110 K). Taking into account the energy of the Cr^3+^ characteristic emission (1.79 eV)^19^ and the corresponding Tanabe-Sugano diagram for *d*^3^ systems^46^, the minimum crystal field value (*Dq*) necessary to observe the *R*-lines is about 1.8 eV. On the other hand, Urusov and Taran^47^ reported the evolution of 10*Dq* as a function of the Cr–O distance (*d*_Cr-O_) for a large number of compounds with octahedral coordinated Cr^3+^ ions obtaining the relation 10*Dq ∝* (*d*_Cr-O_)^−5^, revealing the high sensitivity of the CF to the local environment. In accordance to our calculated Cr–O distances (1.99–2.00 Å) and applying a correction factor of 0.99 to arrange the calculations to experimental TiO_2_ lattice parameters, the 10*Dq* parameter could vary from 2.05 to 2.12 eV. Therefore, according to the Tanabe-Sugano diagrams, it should be possible to observe the *R*-lines in Cr-doped TiO_2_. However, the energy separation between Ti^3+^ (0.9 eV below E_F_) and Cr^3+^ (2.6 eV below E_F_) defect states obtained from the VB spectrum [Fig. 7(e)] is about 1.7 eV. Therefore, the proximity of V_O_ plays an important role on the recombination process of Cr^3+^ ions in rutile TiO_2_. As the Cr concentration increases the charge neutrality can be achieved by creating Cr^4+^ defects or by inducing V_O_ that, according to our results, in the most stable structures tends to transfer electrons to the Cr atoms resulting in an increment of V_O_^2+^ defects.

## Conclusion

In summary, single crystalline Cr doped TiO_2_ micro-tubes have been employed as model material to success-fully compare theoretical simulations and experimental results on the Cr doping of rutile TiO_2_. As a result, a deeper comprehension on the Cr incorporation in the rutile structure has been obtained. Aspects regarding the incorporation of chromium by forming a complex defect with two Cr atoms and one O vacancy (2Cr_Ti_ + V_O_) have been elucidated. The electrons from the oxygen vacancy tend to be localized at the *t*_2*g*_ states of the Cr ions in order to reach the stable oxidation state of Cr^3+^. These results, showing that oxygen defects play a crucial role in the stabilization of Cr^3+^ in the rutile lattice, have been confirmed both theoretically and experimentally, as the separation between the theoretical states due to Ti(3*d*) and Cr(3*d*), calculated by the pDOS, is in agreement with the one obtained experimentally by XPS resonant measurements. Moreover Cr effects on the crystal field and tetragonal distortion have been studied both from DFT simulations as well as by fitting experimental XAS measurements. The results indicate a decrease in the value of the 10*Dq* parameter and the tetragonal distortion |*δ*| in samples with Cr content lower than 3 cat.%, whereas these parameters increase for samples with higher Cr concentrations. Cr doping of rutile TiO_2_ leads to the generation of an energy level 0.55 eV over the VBM of the TiO_2_ as obtained by DFT simulations, which is in agreement to the Cr-resonant XPS measurements of the VB. These Cr related shallow levels behave competitively with the Ti^3+^ defect related level, as measured by luminescence and XPS.

## Electronic supplementary material

Supplementary Information
