# Supplementary material for: Understanding the effects of Cr doping in rutile TiO2 by DFT calculations and X-ray spectroscopy
Source: Sci Rep. 2018 Jun 7;8:8740. doi: 10.1038/s41598-018-26728-3 (PMC5992178; doi:10.1038/s41598-018-26728-3)

Supplementary Information

Understanding the effects of Cr doping in rutile TiO_2 by DFT calculations and X-ray spectroscopy

G. Cristian Vásquez,* David Maestre, Ana Cremades, Julio Ramírez-Castellanos, Elena Magnano, Silvia Nappini and Smagul Zh. Karazhanov

*Corresponding Author:

G. Cristian Vásquez
E-mail: gc.vasquez@ucm.es
Current address: Centre for Materials Science and Nanotechnology, University of Oslo, N-0318 Oslo, Norway

Figure S1: Calculated DOS using the HSE06 functional for (a) Ionized (from 2Cr +V_O defect) and neutral Cr defect in TiO_2, and (b) comparison between HSE06 and PAW-PBE functionals for the 2Cr +V_O defect.

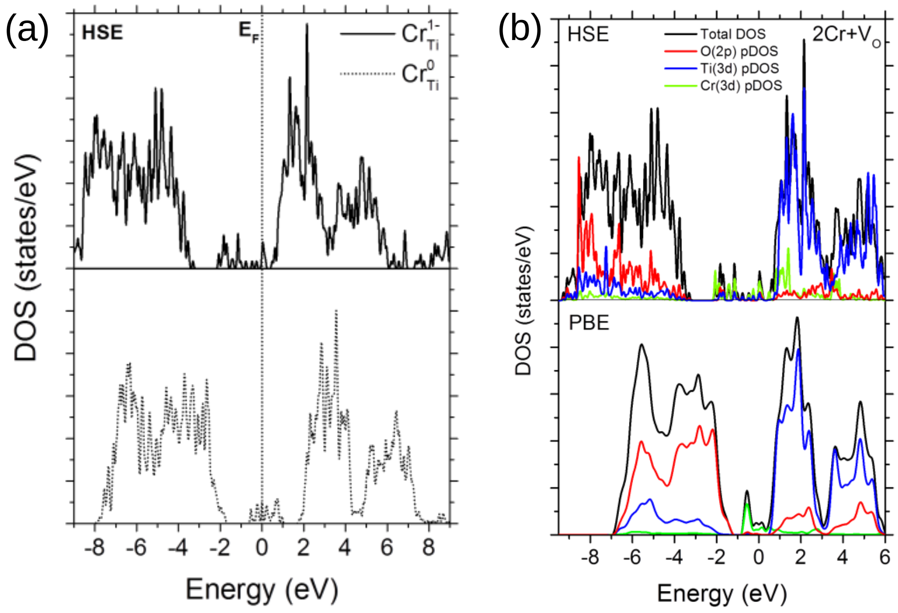

Supplement: Supplementary file 1 — Supplementary Information [file 41598_2018_26728_MOESM1_ESM.pdf]
